# Heartland Virus Disease — United States, 2012–2013

**Published:** 2014-03-28

**Authors:** Daniel M. Pastula, George Turabelidze, Karen F. Yates, Timothy F. Jones, Amy J. Lambert, Amanda J. Panella, Olga I. Kosoy, Jason O. Velez, Marc Fischer, J. Erin Staples

**Affiliations:** 1 EIS officer, CDC; 2 Missouri Department of Health and Senior Services; 3 Tennessee Department of Health; 4 Arboviral Diseases Branch, National Center for Emerging and Zoonotic Diseases, CDC

Heartland virus is a newly identified phlebovirus that was first isolated from two northwestern Missouri farmers hospitalized with fever, leukopenia, and thrombocytopenia in 2009 (1). Based on the patients’ clinical findings and their reported exposures, the virus was suspected to be transmitted by ticks. After this discovery, CDC worked with state and local partners to define the ecology and modes of transmission of Heartland virus, develop diagnostic assays, and identify additional cases to describe the epidemiology and clinical disease. From this work, it was learned that Heartland virus is found in the Lone Star tick (*Amblyomma americanum*) (Figure) (2). Six additional cases of Heartland virus disease were identified during 2012–2013; four of those patients were hospitalized, including one with comorbidities who died.

A confirmed case of Heartland virus disease was defined as a clinically compatible illness in a person with laboratory evidence of recent Heartland virus infection. A clinically compatible illness was defined as fever (≥100.4°F [≥38.0°C]), leukopenia (white blood cell count <4,500 cells/mm^3^; normal range = 4,500–12,000 cells/mm^3^), and thrombocytopenia (platelet count <150,000/mm^3^; normal range = 150,000–400,000/mm^3^) without a more likely clinical explanation. Evidence of recent Heartland virus infection included 1) detection of viral RNA by reverse transcriptase–polymerase chain reaction on blood or tissue or 2) a ≥4-fold rise in virus-specific plaque reduction neutralization antibody titers between acute and convalescent serum specimens.

During 2012–2013, six confirmed Heartland virus disease cases were identified; five patients were Missouri residents, and one was a Tennessee resident. All patients were men aged ≥50 years (median = 58 years; range = 50–80 years). Patients had symptom onset during May to September (three cases in May, one in July, and two in September). All of the patients had fever, thrombocytopenia, and leukopenia when first evaluated. Of the five patients whose acute symptoms were systematically recorded, all reported fatigue and anorexia, and four reported headache, nausea, myalgia, or arthralgia. Four of the patients were hospitalized. One patient with multiple comorbidities died. All of the patients reported spending several hours per day outside (e.g., working, walking, doing yard work, hunting, or hiking), and five of the six patients reported tick bites in the 14 days preceding their illness onset.

No vaccine or medication is available to prevent or treat Heartland virus disease. Because the virus likely is transmitted through infected ticks or other arthropods, prevention depends on using insect repellents, wearing long sleeves and pants, avoiding bushy and wooded areas, and performing tick checks after spending time outdoors. Health-care providers should consider Heartland virus testing in patients who develop fever, leukopenia, and thrombocytopenia without a more likely explanation and who have tested negative for *Ehrlichia* and *Anaplasma* infection or have not responded to doxycycline therapy (3). Questions regarding Heartland virus testing should be directed to state health departments or to the CDC Arboviral Diseases Branch (telephone: 970-221-6400).

## Figures and Tables

**FIGURE:**
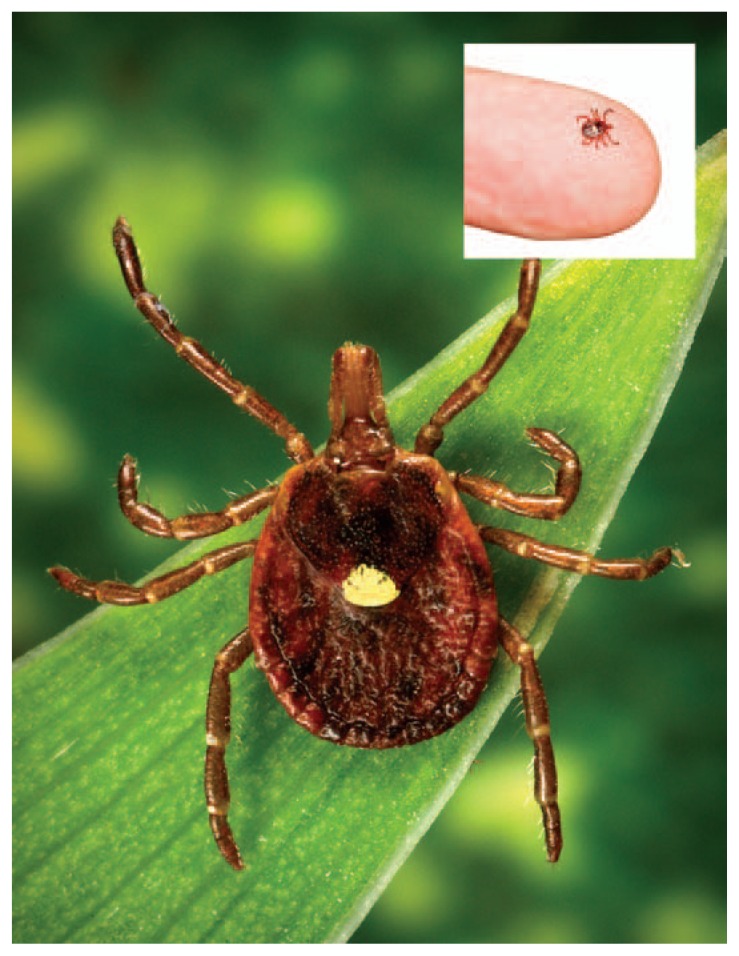
Heartland virus has been found in the Lone Star tick (*Amblyomma americanum*)
